# Mitotic phosphorylation of tumor suppressor DAB2IP maintains spindle assembly checkpoint and chromosomal stability through activating PLK1-Mps1 signal pathway and stabilizing mitotic checkpoint complex

**DOI:** 10.1038/s41388-021-02106-8

**Published:** 2021-11-13

**Authors:** Lan Yu, Yue Lang, Ching-Cheng Hsu, Wei-Min Chen, Jui-Chung Chiang, Jer-Tsong Hsieh, Michael D. Story, Zeng-Fu Shang, Benjamin P. C. Chen, Debabrata Saha

**Affiliations:** 1 Department of Radiation Oncology, University of Texas Southwestern Medical Center, Dallas, TX 75390 USA; 2 Suzhou Digestive Diseases and Nutrition Research Center, The Affiliated Suzhou Hospital of Nanjing Medical University, Suzhou, 215008 China; 3 State Key Laboratory of Radiation Medicine and Protection, School of Radiation Medicine and Protection, Medical College of Soochow University, Collaborative Innovation Center of Radiation Medicine of Jiangsu Higher Education Institutions, Soochow University, Suzhou, 215123 China; 4 Department of Urology, University of Texas Southwestern Medical Center, Dallas, TX 75390 USA; 5 Simmons Comprehensive Cancer Center, University of Texas Southwestern Medical Center, Dallas, TX 75390 USA; 6 Department of Oncology, National Taiwan University Hospital, National Taiwan University College of Medicine, Taipei, 10048 Taiwan

**Keywords:** Oncogenes, Mitosis

## Abstract

Chromosomal instability (CIN) is a driving force for cancer development. The most common causes of CIN include the dysregulation of the spindle assembly checkpoint (SAC), which is a surveillance mechanism that prevents premature chromosome separation during mitosis by targeting anaphase-promoting complex/cyclosome (APC/C). DAB2IP is frequently silenced in advanced prostate cancer (PCa) and is associated with aggressive phenotypes of PCa. Our previous study showed that DAB2IP activates PLK1 and functions in mitotic regulation. Here, we report the novel mitotic phosphorylation of DAB2IP by Cdks, which mediates DAB2IP’s interaction with PLK1 and the activation of the PLK1-Mps1 pathway. DAB2IP interacts with Cdc20 in a phosphorylation-independent manner. However, the phosphorylation of DAB2IP inhibits the ubiquitylation of Cdc20 in response to SAC, and blocks the premature release of the APC/C-MCC. The PLK1-Mps1 pathway plays an important role in mitotic checkpoint complex (MCC) assembly. It is likely that DAB2IP acts as a scaffold to aid PLK1-Mps1 in targeting Cdc20. Depletion or loss of the Cdks-mediated phosphorylation of DAB2IP destabilizes the MCC, impairs the SAC, and increases chromosome missegregation and subsequent CIN, thus contributing to tumorigenesis. Collectively, these results demonstrate the mechanism of DAB2IP in SAC regulation and provide a rationale for targeting the SAC to cause lethal CIN against DAB2IP-deficient aggressive PCa, which exhibits a weak SAC.

## Introduction

Chromosome instability (CIN), generated from increased chromosome missegregation in mitosis, is a driving force of human cancer and is, often correlated with poor prognosis, metastasis, and therapeutic resistance in various cancers [1, 2]. The spindle assembly checkpoint (SAC) is a signaling cascade that prevents chromosome missegregation and CIN by stalling mitotic progression until all chromosomes are properly attached to microtubules emanating from the opposite mitotic spindle poles [3]. The SAC functions by targeting the anaphase-promoting complex/cyclosome (APC/C), a multi-subunit E3 ubiquitin ligase that is critical for the degradation of key mitotic regulators, the metaphase to anaphase transition, and mitotic exit [3]. In response to unattached kinetochores, the SAC prevents the binding of substrates with APC/C-Cdc20 E3 ligase by enhancing the assembly of the mitotic checkpoint complex (MCC), which is a tetrameric protein complex consisting of BubR1, Bub3, Mad2, and Cdc20 [3]. Monopolar spindle 1 (Mps1) kinase is a master regulator for MCC formation that facilitates the kinetochore localization of several key checkpoint proteins, including Bub1, BubR1, Mad1, and Mad2 [4–6]. Mps1 phosphorylates MELT motifs of kinetochore protein KNL1 to create a docking platform for the Bub3-Bub1 and Bub3-BubR1 complexes [5, 7, 8]. Furthermore, Mps1-mediated phosphorylation of Mad1 significantly accelerates the conversion of Mad2 from its inactivated “open” form to its activated “closed” form, after which c-Mad2 interacts with Cdc20, inducing MCC assembly and ACP/C inhibition [9, 10]. The activation of Mps1 relies on its intermolecular autophosphorylation [11]. In addition, polo-box kinase 1 (PLK1), a key regulator involved in numerous steps during mitotic progression, can also phosphorylate Mps1 on its autophosphorylation sites and enhance its catalytic activity, which contributes to the recruitment of SAC components to kinetochores [12]. Protein phosphatase 1–mediated dephosphorylation of Mps1 is required for its inactivation and for timely SAC silencing [13]. In addition to its phosphorylation status, Mps1’s protein abundance is also regulated throughout the cell cycle. Mps1 can be ubiquitinated by the APC/C E3 enzyme at its D-box motifs, and following proteolytic degradation to ensure efficient SAC inactivation. Overexpression of Mps1 is sufficient to reactivate the SAC during anaphase [14]. When all chromosomes are bi-oriented, the MCC must be disassembled to activate APC/C-Cdc20 and mitotic exit. Several proteins are implicated in MCC disassembly, such as p31 (comet) [15, 16] and CUEDC2 [17]. Cyclin-dependent kinase 1 (Cdk1)-mediated phosphorylation of these proteins is also important for their function in MCC disassembly [17, 18]. In addition, the APC/C mediated Cdc20 autoubiquitylation and degradation in mitosis contributes to timely APC/C-MCC disassembly and allows APC/C to form a complex with the newly synthesized Cdc20 [19]. Recent studies demonstrated that the dynamic assembly and disassembly of APC/C-MCC occurs continuously, even in the presence of the SAC. This dynamic character of MCC assembly and disassembly is a key feature that allows rapid checkpoint silencing when the SAC turns off [20–22].

DOC-2/DAB2 interactive protein (DAB2IP), a potential tumor suppressor, is often observed to be repressed by epigenetic silencing in a variety of malignant tumors [23]. DAB2IP acts as a scaffold protein that maintains cellular homeostasis through modulating a variety of biologic events, including cell growth, apoptosis, survival, and epithelial–mesenchymal transition. Mechanistically, DAB2IP inhibits the PI3K-Akt pathway and activates ASK1-JNK-mediated apoptosis [24, 25]. P53 missense mutants (mutp53) interact with DAB2IP and inhibit its function in stimulating ASK1-JNK–mediated apoptosis in response to inflammatory cytokines [26]. DAB2IP can also suppress the stemness of cancer cells by impairing CD117 transcription [27]. Recently, several studies revealed that the loss of DAB2IP expression is associated with therapeutic resistance in cancer cells. Downregulating DAB2IP induces radioresistance in prostate cancer (PCa) cells by enhancing the repair of damaged DNA and maintaining a robust G2 cell cycle checkpoint [28, 29]. DAB2IP can interact with PARP-1 and contribute to E3 ligase–mediated ubiquitylation and degradation of PARP-1, which increases the radiosensitivity of renal cell carcinoma cells [30]. The expression of DAB2IP also restores the chemoresistance of castration-resistant PCa cells by suppressing the Egr-1/Clusterin pathway [31]. We previously found that DAB2IP participates in mitosis regulation including SAC activation and the stabilization of kinetochore-microtubule attachments. Loss of DAB2IP increases chromosome missegregation and CIN [32]. However, the underlying molecular mechanism for the regulation of the SAC-related signal pathway needs to be further clarified. Here, we report that DAB2IP can be phosphorylated by Cdks at its Thr531 and Thr546 sites during mitosis, and this DAB2IP phosphorylation is required to activate PLK1 and its downstream target Mps1 kinase during mitosis. Meanwhile, DAB2IP binds with Cdc20 and blocks Cdc20 ubiquitylation and degradation, thus maintaining MCC stability during prometaphase and SAC activation. Our findings highlight a new mechanism by which DAB2IP contributes to the SAC and mitigates the aggressiveness of PCa.

## Results

### DAB2IP depletion accelerates the exit of PCa cells from mitosis and promotes the disassembly of APC/C-MCC

**Fig. 1:**
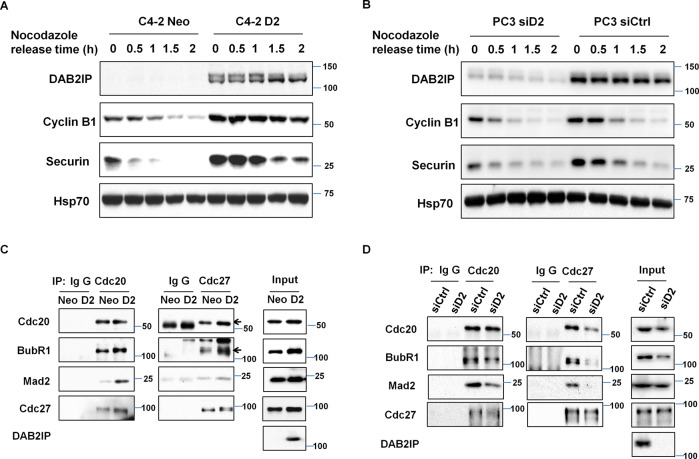
DAB2IP prevents premature mitotic exit and maintains MCC stability. DAB2IP-proficient and -deficient C4-2 (C4-2 D2 and C4-2 Neo cells) (**A**) and PC3 (PC3 siCtrl cells and siD2) (**B**) cells were synchronized in prometaphase using nocodazole (50 ng/ml) and then released into fresh media. Cells were collected at the indicated times after release. The expression of DAB2IP, Cyclin B1, Securin, and HSP70 were detected via immunoblotting. Cdc20 and APC/C complex were immunoprecipitated from nocodazole-blocked C4-2 D2 and Neo (**C**), and from DAB2IP-knockdown PC3 and its control (**D**) prometaphase cells lysates. The amounts of Mad2 and BubR1 binding with Cdc20 and Cdc27 were determined by immunoblotting.

Our previous study revealed that the loss of DAB2IP impairs mitotic arrest induced by a microtubule-damaging agent, which suggests a potential role for DAB2IP in SAC maintenance [32]. To elucidate the function of DAB2IP in checkpoint inactivation, we examined the degradation of cyclin B1 and securin in PCa cells with or without expression of DAB2IP after release from nocodazole-induced mitotic arrest. When compared with DAB2IP-deficient cells (C4-2 Neo and PC3 siD2), DAB2IP-expressing PCa cells (C4-2 D2 and PC3 siCtrl) exhibited dramatically stabilized cyclin B1 and securin (Fig. 1A, B). Flow cytometry analysis also supported the conclusion that DAB2IP slows down the release of PCa cells from mitotic arrest (Supplementary Fig. S1A). Because APC/C activation is determined by its association pattern, we further questioned whether DAB2IP alters mitotic exit via affecting the interaction status of the APC/C-MCC complex. Nocodazole synchronized, prometaphase C4-2 D2 and C4-2 Neo cells were immunoprecipitated by using anti-Cdc20 and anti-Cdc27 antibodies. The amount of Mad2 and BubR1 bound to Cdc20 and Cdc27 was elevated in DAB2IP-expressing C4-2 cells (Fig. 1C). Consistently, knockdown of DAB2IP in PC3 or HeLa cells also decreased the accumulation of Mad2 and BubR1 on Cdc20 and Cdc27 (Fig. 1D and Supplementary Fig. S1B). When cells were released from nocodazole-blocked mitosis, the interaction of Mad2 with Cdc20 was more stabilized in C4-2 D2 cells than in C4-2 Neo cells (Supplementary Fig. S1C).

### DAB2IP interacts with Cdc20 and inhibits the ubiquitylation and degradation of Cdc20 in prometaphase

**Fig. 2:**
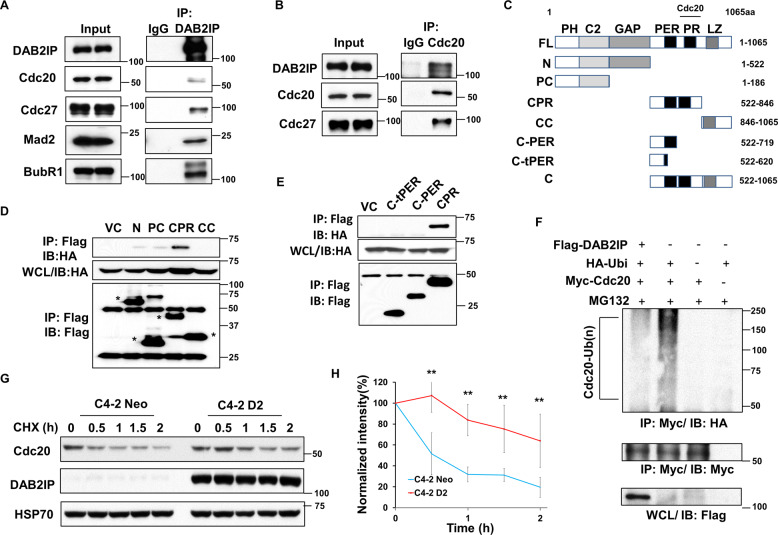
DAB2IP interacts with Cdc20, and inhibits the ubiquitylation mediated degradation of Cdc20 in prometaphase. **A**, **B** C4-2 D2 cells were synchronized at prometaphase and the mitotic cells were collected by the shake-off method. Cells lysates were immunoprecipitated with anti-DAB2IP (**A**) or anti-Cdc20 (**B**) or IgG antibodies, and the interaction with Cdc20, DAB2IP, Cdc27, BubR1, and Mad2 were analyzed by immunoblotting. **C** Schema of different truncated domains of DAB2IP. **D**, **E** Various truncated cDNA constructs of DAB2IP were co-transfected with HA-Cdc20 into HeLa cells. HeLa cells were then synchronized at prometaphase and the mitotic cells were collected by the shake-off method. Cells lysates were immunoprecipitated with anti-Flag with antibody. The signal of HA and Flag were determined by immunoblotting. **F** Myc-Cdc20 and HA-Ubi were co-transfected with Flag-DAB2IP or empty vector in HeLa cells. Cells were harvested at 6 h after MG132 (10 μM) treatment. Cdc20 was immunoprecipitated using anti-Myc antibody. Co-IP products were analyzed by immunoblotting using anti-HA and anti-Myc antibodies. Equal loading of whole cell lysates were subjected to immunoblotting by anti-Flag antibody. **G** C4-2 D2 and Neo cells were arrested at prometaphase and treated with cycloheximide (0.1 mg/ml) and nocodazole (50 ng/ml) for the indicated times. The degradation rate of Cdc20 was determined by immunoblotting. **H** Mean Cdc20 protein levels in cycloheximide-treated C4-2 D2 and Neo cells (*n* = 4; error bars, s.e.m.). The intensity was normalized to HSP70 levels.

Furthermore, we tested whether DAB2IP could directly bind with the MCC during mitosis. C4-2 D2 cells were enriched in prometaphase by using nocodazole and mitotic cells were harvested by shake-off. The synchronized M-phase C4-2 D2 cell lysates were then subjected to co-immunoprecipitation (co-IP) analysis. Cdc27, the core component of APC/C, co-activator Cdc20, and other factors of the MCC, such as Mad2 and BubR1, were precipitated by DAB2IP antibodies (Fig. 2A). DAB2IP was consistently precipitated by the anti-Cdc20 antibody (Fig. 2B). DAB2IP also formed a complex with Cdc20, Cdc27, BubR1, and Mad2 in mitotic HeLa cells (Supplementary Fig. S2A). The Flag-DAB2IP expression construct or the empty vector were co-transfected with HA-Cdc20, then subjected to co-IP using an anti-Flag antibody. The results showed that HA-Cdc20 can be immunoprecipitated by anti-Flag antibody (Supplementary Fig. S2B). To further characterize the domain of DAB2IP that mediates its interaction with Cdc20, we co-transfected Flag-tagged truncated DAB2IP expression constructs, which cover various regions of DAB2IP, with HA-Cdc20 in 293T cells, then performed co-IP using an anti-Flag antibody. The data indicated that the CPR domain of DAB2IP interacts with Cdc20 (Fig. 2C, D). We further narrowed down the truncated domains of DAB2IP and found that loss of the PR region eliminates the interaction between DAB2IP and Cdc20 (Fig. 2E). Because this CPR region includes a proline-rich domain, which is important for mediating protein-protein interaction [24, 25], we co-transfected a DAB2IP AAA mutant (eight prolines in the PR domain were replace by alanine [24], Supplementary Fig. S2C) with Cdc20, then tested the immunoprecipitation. Our results showed that the AAA mutant weakened the interaction between DAB2IP and Cdc20, but did not abolish its binding to Cdc20 (Supplementary Fig. S2D). Since Cdc20 autoubiquitylation by APC/C is required for MCC disassembly and SAC silencing, we then tested the effect of DAB2IP on the ubiquitylation level of Cdc20 during prometaphase. HeLa cells were transfected with Myc-Cdc20, Flag-DAB2IP (or Flag empty vector), and HA-Ubi expression vector, and synchronized in prometaphase via nocodazole followed by MG132. Results showed that Cdc20 ubiquitylation was much weaker in DAB2IP-expressing cells than in DAB2IP-deficient cells (Fig. 2F), a finding confirmed in 293T cells (Supplementary Fig. S2E). It has been reported that Cdc20 is continuously synthesized and degraded during prometaphase [33]. Consistent with lower ubiquitin levels, the proteolysis rate of Cdc20 was reduced in DAB2IP-expressing C4-2 cells (Fig. 2G, H). These findings indicate that DAB2IP might be required to maintain a robust SAC by mitigating the ubiquitylation and subsequent degradation of Cdc20 during prometaphase.

### DAB2IP stabilizes Mps1 protein and promotes the kinase activity of Mps1

**Fig. 3:**
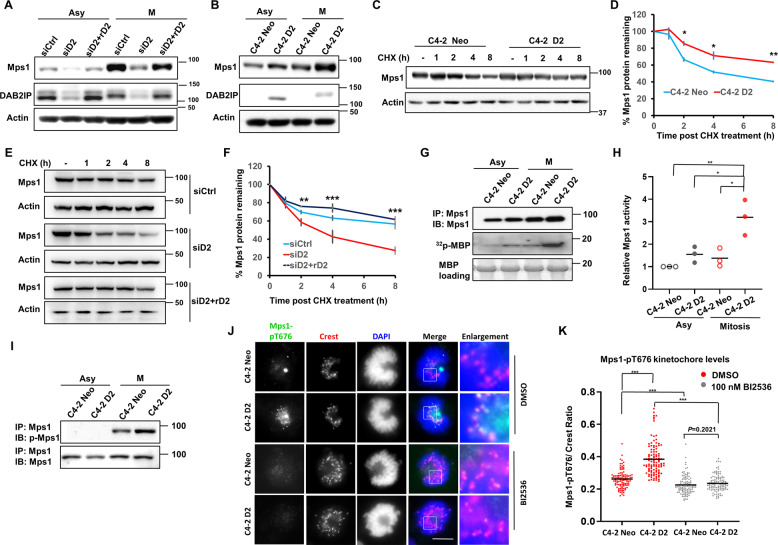
DAB2IP stabilizes Mps1 protein and promotes the kinase activity of Mps1. Paired PC3 and DAB2IP rescued PC3 (rD2) (**A**), and C4-2 (**B**) cells were treated with nocodazole (50 ng/ml) for 16 h. Mitotic (M) and asynchronous (Asy) cells were analyzed by immunoblotting with anti-Mps1, anti-DAB2IP, and anti-Actin antibodies. **C** C4-2 D2 and Neo cells were treated with cycloheximide (0.1 mg/ml) for the indicated times. The degradation rate of Mps1 was determined by immunoblotting. **D** Mean Mps1 protein levels in cycloheximide-treated C4-2 D2 and Neo cells (*n* = 3; ^*^P < 0.05 and ^**^P < 0.01 as compared with Neo cells.). The intensity was normalized to Actin levels. **E** PC3 cells were transfected with siRNA against DAB2IP or transfected along with siRNA-resistant DAB2IP plasmid (rD2) to rescue the expression. Paired PC3 and DAB2IP rescued PC3 (rD2) cells were treated with cycloheximide for the indicated times. The degradation rate of Mps1 was determined by immunoblotting. **F** Mean Mps1 protein levels in cycloheximide-treated DAB2IP-deficient, DAB2IP-rescued and control PC3 cells (*n* = 3; ^**^P < 0.01, ^***^P < 0.001 as compared with control cells.). The intensity was normalized to Actin levels. **G** Mitotic (M) and asynchronous (Asy) C4-2 Neo and C4-2 D2 cells were analyzed for Mps1 activity by the in vitro kinase assay using MBP as the substrate. Phosphorylation of MBP was visualized by autoradiography. **H** Autoradiography of MBP was normalized to Mps1 protein level, and to Mps1 activity in asynchronous C4-2 Neo cells, then graphed for three independent experiments (*n* = 3; ^*^P < 0.05 and ^**^P < 0.01 as compared with mitotic C4-2 D2 cells.). **I** Mitotic (M) and asynchronous (Asy) C4-2 Neo and C4-2 D2 cell lysates were immunoprecipitated with an anti-Mps1 antibody followed by immunoblotting with anti-Mps1-pThr676 and anti-Mps1 antibodies. **J**, **K** Asynchronously growing C4-2 Neo and D2 cells were incubated with or without PLK1 inhibitor (BI2536) for 2 h, then stained with anti-Mps1-pT676 antibody. Immunofluorescence staining (**J**) showing kinetochore levels of Mps1-pT676, **K** Quantification of Mps1-pT676 levels (normalized by Crest) on prometaphase kinetochores in paired C4-2 cells (*n* = 30–35 cells, ^***^P < 0.001).

As described previously, Mps1 is essential for APC/C-MCC assembly. Therefore, we also analyzed Mps1 expression in DAB2IP-proficient and -deficient PCa cells. As shown in Fig. 3A, the Mps1 protein level is much lower in DAB2IP-depleted PC3 cells than in control cells and was reversed by expressing siRNA-resistant DAB2IP protein. Consistently, the expression of DAB2IP in C4-2 cells (C4-2 D2) caused a marked increase in the expression of Mps1 (Fig. 3B). We assessed the influences of DAB2IP on the stability of Mps1 in DAB2IP-proficient and -deficient PCa cells. Following treatment with cycloheximide (CHX), PC3 and C4-2 cells were harvested at indicated times to detect Mps1 protein levels. As expected, PCa cells expressing DAB2IP significantly prolonged the half-life of Mps1 protein compared to cells without DAB2IP (Fig. 3C–F). Moreover, the proteasome inhibitor MG132 markedly increased the Msp1 protein level in DAB2IP-deficient C4-2 Neo cells (Supplementary Fig. S2F). In addition, the activity of Mps1 is important for the progression of mitosis. To further validate the role of DAB2IP in Mps1 activity, we immunoprecipitated endogenous Mps1 from asynchronous and nocodazole-arrested mitotic C4-2 D2 and C4-2 Neo cells for in vitro kinase assay using myelin basic protein (MBP) as the substrate. The data showed that Mps1-mediated MBP phosphorylation was dramatically enhanced in C4-2 D2 cells as compared to C4-2 Neo cells (Fig. 3G, H). We immunoprecipitated the Mps1 protein from asynchronous and nocodazole-arrested mitotic DAB2IP-proficient and -deficient PCa cells, and we assessed the autophosphorylation of Mps1 at its T676 site and the total protein. As shown in Fig. 3I, autophosphorylated Mps1 levels were elevated in DAB2IP-expressing C4-2 D2 cells. These results demonstrated that DAB2IP stabilizes Mps1 and promotes the activation of Mps1. To further support this conclusion, we detected Mps1 activity at kinetochores by immunofluorescent staining using anti-Mps1-pT676 antibody. DAB2IP expression significantly increased the presence of phosphorylated Mps1 at kinetochores. Our previous study showed that DAB2IP promotes PLK1 activity [32], which can facilitate Mps1 activation [12]. Here, we found that inhibiting PLK1 suppressed the phosphorylation of Mps1-T676 at kinetochores. More importantly, PLK1 inhibitor totally abolished the differences in phosphorylated Msp1-T676 induced by DAB2IP expression (Fig. 3J, K), which indicates that DAB2IP might regulate Mps1 activity in a PLK1-mediated manner.

### DAB2IP-deficient prostate cancer cells are sensitive to Mps1 inhibitor

**Fig. 4:**
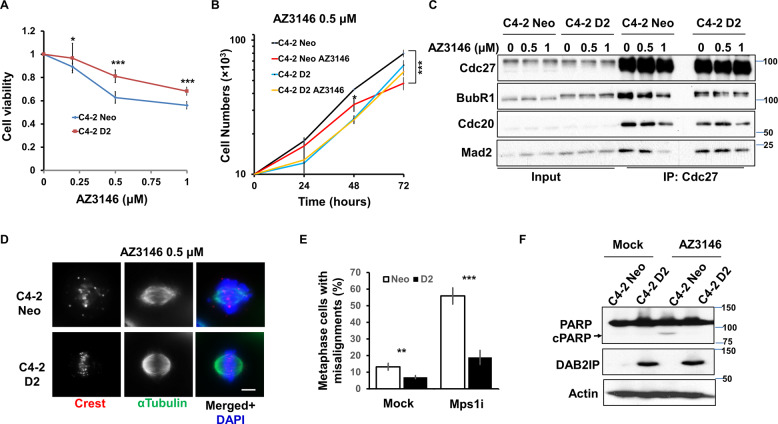
DAB2IP-deficient prostate cancer cells are sensitive to Mps1 inhibitor. **A** Paired C4-2 cells were exposed to indicated concentrations of AZ3156, a specific Mps1 inhibitor, for 48 h and MTT assay was performed. Results obtained were from three independent experiments (means ± SD; ^***^P < 0.001 and ^*^P < 0.05 as compared with Neo cells). **B** 1 × 10^4^ cells were seeded in 35 mm^2^ dishes at day 0, cells were treated with or without AZ3156 (0.5 μM) and cells were counted to determine cell proliferation rates at indicated days (1, 2, and 3 days) (means ± SD; ^*^P < 0.05, ^***^P < 0.001 as compared with untreated cells). **C** C4-2 D2 and Neo cells were arrested at prometaphase and treated with 0.5 and 1 μM of AZ3156 along with nocodazole for an additional 2 h. APC/C complex was immunoprecipitated from C4-2 D2 and Neo cells lysates treated or untreated with Mps1 inhibitor. The amounts of Mad2, BubR1, and Cdc20 binding with Cdc27 were determined by immunoblotting. **D** C4-2 Neo and D2 cells were treated with 10 μM MG132 for 4 h, then treated with or without 0.5 μM AZD3156 along with MG132 for an additional 2 h. Chromosome alignment was determined by immunofluorescent staining using anti-α-tubulin and Crest antibody; DNA was visible by DAPI. Scale bars are 5 μm. **E** Proportion of cells with chromosome-missegregation and misalignment in C4-2 Neo and C4-2 D2 cells treated with or without AZD3156 are presented as the mean and SD from three independent experiments (^**^P < 0.01, ^***^P < 0.001 as compared with Neo cells). **F** Cells were exposed to 1 μM of AZD3156 for 24 h and subjected to immunoblotting with anti-PARP, anti-DAB2IP, and anti-Actin antibodies.

DAB2IP loss results in PCa resisting common clinically used chemotherapeutic drugs. Mps1 has emerged as a potential therapeutic target for cancer therapy. Here, we found that DAB2IP-deficient PCa cells exhibit a higher sensitivity toward Mps1 inhibitor (AZ3146) at a lower dosage than DAB2IP-proficient PCa cells do (Fig. 4A). The 0.5 μM of AZ3146 significantly inhibited C4-2 Neo cell growth but had almost no influence on C4-2 D2 cell growth (Fig. 4B). Because of the essential role of Mps1 in MCC formation, we determined the integrity of the APC/C-MCC complex upon Mps1 inhibitor by co-IP experiments. AZ3146 (1 μM) disrupted the interaction between APC/C component Cdc27 and the MCC complex (BubR1, Cdc20, and Mad2) in C4-2 Neo cells, whereas APC/C-MCC stability was only slightly affected in C4-2 D2 cells in response to the same dosage of Mps1i (Fig. 4C). Mps1 facilitates the alignment of chromosomes on the metaphase plate. As expected, misaligned chromosomes were more numerous in C4-2 Neo cells than in the C4-2 D2 cells (Fig. 4D, E) in response to the same dosage of Mps1i. Aberrant mitosis is associated with apoptotic cell death. We noticed that PARP-1 cleavage was greater in C4-2 Neo cells than in C4-2 D2 cells in response to a low dosage of Mps1i (Fig. 4F). The study revealed that DAB2IP-deficient PCa cells were more sensitive to a proper concentration of Mps1 inhibitor AZ3146.

### DAB2IP is phosphorylated by Cdks on Thr531 and Thr546 during mitosis

**Fig. 5:**
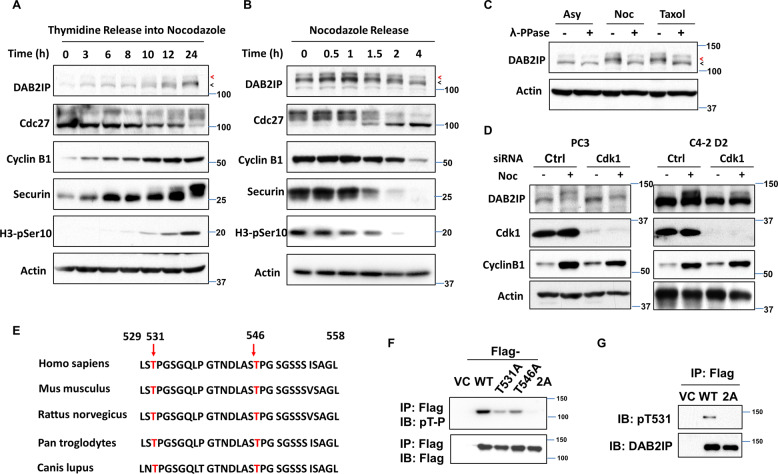
Cdk1 mediates phosphorylation of DAB2IP on its Thr531 and Thr546 sites in mitosis. **A** Thymidine-synchronized PC3 cells were released into normal medium containing nocodazole (50 ng/ml) and harvested at the indicated time points after release. The expression of DAB2IP, Cdc27, cyclin B1, securin, and phosphorylation of histone 3 on Ser10 were determined by using immunoblotting. **B** Mitotically arrested PC3 cells were released for cell cycle reentry and harvested at the indicated time points after release. The expression of DAB2IP, Cdc27, cyclin B1, securin, and phosphorylation of histone 3 on Ser10 were determined by using immunoblotting. **C** Mitotically arrested (nocodazole- or paclitaxel-induced) and asynchronous PC3 cells lysates were treated with or without λ-PPase. The expression pattern of DAB2IP was presented by using immunoblotting. **D** DAB2IP-proficent PC3 and C4-2 D2 cells were transfected with siRNA against *Cdk1* or control siRNA, and 24 h after transfection, cells were treated with nocodazole for an additional 16 h. The expression of DAB2IP, Cdk1, cyclin B1 and Actin in mitotically arrested and asynchronous cells was determined by immunoblotting. **E** Sequence alignment of the DAB2IP proteins from different species (Homo sapiens, Mus musculus, Rattus norvegicus, Pan troglodytes, and Canis lupus) around Thr531 and Thr546. **F** Flag-tagged wild-type DAB2IP, DAB2IP T531A, DAB2IP T546A, DAB2IP 2A (T531A/T546A) and empty vector were expressed in HeLa cells, and HeLa cells were synchronized by nocodazole. The wild-type form of DAB2IP and different mutants were immunoprecipitated by anti-Flag (M2) antibody from mitotically arrested cell lysates. Phosphorylations on DAB2IP and its mutants were detected by immunoblotting using an antibody that recognizes Cdk targeting TP motifs (p-TP). The expression of Flag-tagged protein was also determined. **G** Flag-tagged wild-type DAB2IP, DAB2IP 2A (T531A/T546A) and empty vector were expressed in HeLa cells, and HeLa cells were synchronized by nocodazole. The wild-type form of DAB2IP and the 2A mutant were immunoprecipitated by anti-Flag (M2) antibody from mitotically arrested cells lysates. The phosphorylation of DAB2IP on its Thr531 site and the expression of DAB2IP were detected.

As our previous study [32] and Fig. 1A show, DAB2IP protein was detected as multiple bands by immunoblotting. To determine whether the shift band of DAB2IP occurs in mitosis, we synchronized PC-3 cells with a double-thymidine block at the G1/S boundary and released them into medium with nocodazole for blockage at mitosis. The gradual accumulation of cells at mitosis was confirmed by immunoblotting, which showed the increased expression of cyclin B1 and securin and the phosphorylation of histone 3 at its Ser10 site (Fig. 5A). Following entrance into mitosis, the major band of DAB2IP shifted to the high molecular weight position, and the major band moved back when mitotic exit occurred after nocodazole release (Fig. 5B). We also observed DAB2IP’s molecular shift up during mitosis in HeLa and DU145 cells as well as C4-2 D2 cells with exogenously expressed DAB2IP (Supplementary Fig. S3). The lambda protein phosphatase (λ-PPase) treatment promoted shifting of DAB2IP to the lower molecular weight position, which suggests that DAB2IP is phosphorylated during mitosis (Fig. 5C). Cdk1 is essential for driving mitotic progression. We postulated that DAB2IP might be a potent substrate of Cdk1. Consistent with our hypothesis, treatment with RO-3306, a specific inhibitor of Cdk1, and knockdown of Cdk1 by siRNA prevented the phosphorylation band of DAB2IP from appearing in mitotic lysates (Fig. 5D and Supplementary Fig. S4A). Cdk1 exclusively phosphorylates serine (Ser) or threonine (Thr) residues immediately preceding a proline residue (SP/TP motifs). To further determine whether Cdk1 is responsible for DAB2IP phosphorylation, we immunoprecipitated DAB2IP from C4-2 cells lysates in various cell cycle phases. The immunoprecipitated DAB2IP showed strong immunoreactivity with both anti-p-SP and anti-p-TP antibodies. However, the anti-p-TP antibody specifically recognized Flag-DAB2IP proteins from mitotic lysates (Supplementary Fig. S4B). To identify the Cdk1-mediated phosphorylation sites in DAB2IP, we expressed the different Flag-tag fused truncated domains of DAB2IP in HeLa cells and immunoprecipitated them from asynchronous and mitotic cell lysates. We found that the CPR domain, which was reported to interact with PLK1, exhibited immunoreactivity with anti-p-TP antibody (Supplementary Fig. S4C). There are two conserved TP motifs, Thr531 and Thr546, in the CPR region of DAB2IP (Fig. 5E). To determine whether these two sites were phosphorylated during mitosis, we replaced Thr531 and Thr546 with alanine, both separately and simultaneously, and determined the p-TP levels by immunoblotting. Both the DAB2IP-Thr531Ala and the DAB2IP-Thr546Ala mutants exhibited dramatically decreased levels of p-TP immunoreactivity, while the DAB2IP-2A mutant was barely detectable with the anti-p-TP antibody (Fig. 5F), which suggests that Thr531 and Thr546 are major targets for Cdk1-mediated phosphorylation in DAB2IP during mitosis. Phospho-specific antibodies against the DAB2IP Thr531 site were generated and showed a minimal response for the DAB2IP-2A mutant (Fig. 5G). These results demonstrate the Cdk1-mediated phosphorylation of DAB2IP on its Thr531 and Thr546 sites in mitosis.

### Phosphorylation of DAB2IP at its Thr531 and Thr546 sites activates the PLK1-Mps1 signal pathway and enhances APC/C-MCC complex stability

**Fig. 6:**
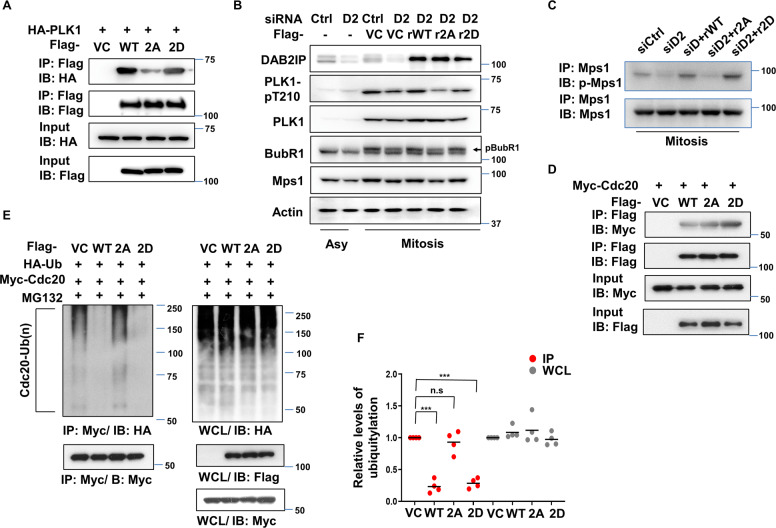
Phosphorylation of DAB2IP at its Thr531 and Thr546 sites activates the PLK1-Mps1 signal pathway and inhibits Cdc20 ubiquitylation in prometaphase. **A** Flag-tagged-DAB2IP, -DAB2IP 2A (T531A/T546A), -DAB2IP 2D (T531D/T546D), empty vector and HA-PLK1 were transfected into HeLa cells. The cells were then treated with 50 ng/ml nocodazole for 16 h and mitotic cells lysates were immunoprecipitated with anti-Flag antibody; the HA signal was examined by immunoblotting. **B** Immunoblotting analysis levels of PLK1-pT210, PLK1, Mps1, BubR1 in mitotically arrested or asynchronous PC3 cells with siRNA-mediated DAB2IP suppression and overexpression of siRNA-resistant DAB2IP (rWT), siRNA-resistant DAB2IP 2A (r2A), and siRNA-resistant DAB2IP 2D (r2D) constructs. **C** PC3 cells with siRNA-mediated DAB2IP suppression and overexpression of siRNA-resistant DAB2IP (rWT), siRNA-resistant DAB2IP 2A (r2A), and siRNA-resistant DAB2IP 2D (r2D) constructs were arrested in mitosis by nocodazole, and shake-off cell lysates were immunoprecipitated by anti-Mps1 antibody. The phosphorylation of Mps1 on its Thr676 site and the amount of Mps1 protein were detected. **D** Flag-tagged-DAB2IP, -DAB2IP 2A (T531A/T546A), -DAB2IP 2D (T531D/T546D), empty vector and Myc-Cdc20 were transfected into HeLa cells. The cells were then treated with 50 ng/ml nocodazole for 16 h and mitotic cells lysates were immunoprecipitated with anti-Flag antibody; the Myc signal was examined by immunoblotting. **E** Myc-Cdc20 and HA-Ubi were co-transfected with Flag-DAB2IP, Flag-DAB2IP 2A (T531A/T546A), Flag-DAB2IP 2D (T531D/T546D) or empty vector in HeLa cells. Cells were harvested at 6 h after MG132 (10 μM) treatment. Cdc20 was immunoprecipitated by using anti-Myc antibody. Co-IP products were analyzed by immunoblotting using anti-HA and anti-Myc antibodies. Equal loadings of whole cell lysates were subjected to immunoblotting by anti-Flag and anti-HA antibodies. **F** The intensity of ubiquitinated proteins was normalized to VC transfected cells, then graphed for four independent experiments (*n* = 4; ^***^P < 0.001 as compared with VC transfected cells.).

Our previous work demonstrated that the CPR region of DAB2IP interacts with the polo-box domain (PBD) of PLK1 [32], which is involved in the activation of Mps1 and plays pivotal roles in mitotic progression. The PBD domain of PLK1 commonly binds to a Cdks prime-phosphorylated substrate and recognizes a consensus sequence of S-pS/pT-P/X, through which PLK1 is recruited to the mitotic apparatus and releases its N-terminal kinase domain to determine the spatiotemporal dynamics of PLK1 during mitosis. Here, we examined whether the Thr531 and Thr546 of DAB2IP mediate DAB2IP’s interaction with PLK1. Thr531 and Thr546 were mutated to Ala (DAB2IP 2A) or Asp (DAB2IP 2D. Co-IP analysis demonstrated that the mutation of these sites into Ala significantly reduced the interaction between DAB2IP and PLK1 during mitosis, whereas substituting these phosphorylation sites with Asp restored their binding (Fig. 6A). The impaired mitotic autophosphorylation of PLK1 at Thr-210 in DAB2IP knockdown PC3 cells was reversed by expressing siRNA-resistant wild-type DAB2IP and DAB2IP 2D proteins, but not by expressing siRNA-resistant DAB2IP 2A mutant (Fig. 6B). We generated the DAB2IP- and DAB2IP 2A-overexpressed stable C4-2 cell lines and also observed that the DAB2IP 2A mutant impairs the mitotic phosphorylation of PLK1 and BubR1 (Supplementary Fig. S5A). More importantly, Mps1 protein levels, the autophosphorylation of Mps1, and the mitotic phosphorylation of BubR1 can be stimulated by wild-type DAB2IP and DAB2IP 2D protein but not by the DAB2IP 2A mutant (Fig. 6B, C).

Next, we tested whether DAB2IP phosphorylation was required for its association with Cdc20. The 2A mutant of DAB2IP exhibited relative interaction efficiency with Cdc20, which suggests that the phosphorylation of DAB2IP at the Thr531 and Thr546 residues might not be necessary for its binding with Cdc20 (Fig. 6D). However, we found that phosphorylated DAB2IP is required to block the ubiquitylation of Cdc20 during prometaphase (Fig. 6E, F). The ubiquitylation of Cdc20 is critical for APC/C-MCC disassociation. Consistent with this, DAB2IP overexpression increased the amount of Cdc20-bound Mad2 in prometaphase cell lysates, whereas DAB2IP 2A overexpression had almost no effect on the interaction between Cdc20 and Mad2 during prometaphase (Supplementary Fig. S5B).

### Phosphorylation of DAB2IP activates SAC and stabilizes k-MT attachment, and suppression of these sites increases chromosomal instability and tumorigenesis in PCa cells

**Fig. 7:**
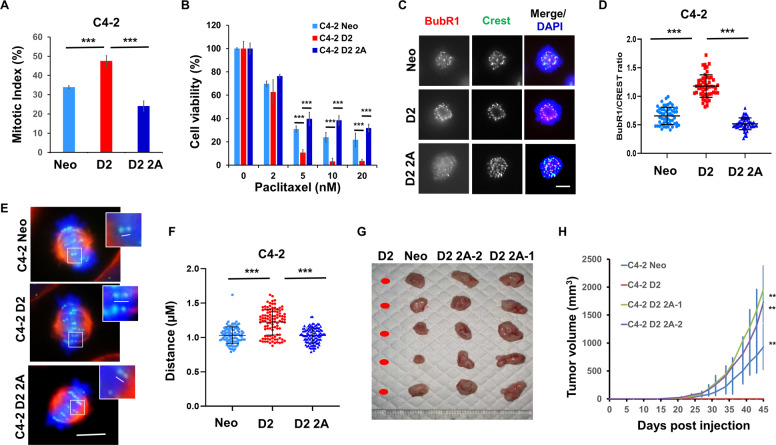
Phosphorylation of DAB2IP at its Thr531 and Thr546 sites promotes SAC activation, k-MT attachment and inhibits PCa tumorigenesis. **A** C4-2 Neo, D2, and D2 2A (T531A/T546A) cells were treated with 50 ng/ml nocodazole for 16 h. Mitotic index was determined by chromosome spread assay (three independent experiments, *n* ≥ 1000 in each group; error bars, s.e.m; ^***^P < 0.001 as compared with Neo and D2 2 A cells). **B** C4-2 Neo, D2, and D2 2A cells were exposed to different concentrations of paclitaxel for 48 h and MTT assay was performed. Results were obtained from three independent experiments (means ± SD; ^***^P < 0.001 as compared with control and D2 2A cells). **C**, **D** Decreased kinetochore localization of BubR1 in phosphoryl-deficient DAB2IP-expressing PCa cells. Representative images showing BubR1 colocalization with kinetochores (Crest) in C4-2 Neo, D2, and D2 2A cells. C4-2 cells were fixed and immunostained with anti-BubR1 and Crest antibodies (**C**). Colocalization of BubR1 with Crest antibody-stained structures (kinetochores) was detected under a fluorescence microscope. Scale bar, 5 μm. Quantification of BubR1 levels (normalized by Crest) on prometaphase kinetochores in C4-2 cells expressing different DAB2IP statuses (**D**). (^***^P < 0.001 as compared with control and D2 2A cells). **E** Inter-distance of paired kinetochores at metaphase in C4-2 Neo, D2, and D2 2A cells. **F** Average inter-kinetochore distances of C4-2 Neo, D2 and D2 2A cells. To quantify inter-kinetochore distances, all images were acquired as Z-stack with 0.4 μm spacing using a ×63 objective. At least 100 kinetochores were analyzed in each cell lines. Error bars represent SD (^***^P < 0.001 as compared with control and D2 2A cells). **G**, **H** The phosphorylation of DAB2IP on its Thr531 and Thr546 is essential to suppress tumor growth in vivo. (*n* = 5; ^**^P < 0.01 as compared with C4-2 D2 cells).

We further investigated the role of phosphorylated DAB2IP in mitotic progression. When compared to parental DAB2IP-deficient C4-2 Neo cells and DAB2IP 2A-expressing cells, wild-type DAB2IP-expressing cells exhibited a much higher percentage of mitotic cells after nocodazole treatment (Fig. 7A). Consistently, we demonstrated that wild-type DAB2IP-expressing cells exhibited higher sensitivity toward paclitaxel treatment than parental DAB2IP-deficient C4-2 Neo cells and DAB2IP 2A-expressing C4-2 cells did (Fig. 7B). The kinetochore localization of BubR1 is associated with SAC maintenance and is affected by DAB2IP. Here, we also found that phosphorylated DAB2IP contributes to the loading of BubR1 onto kinetochores during prometaphase (Fig. 7C, D). The PLK1-BubR1 signal is also essential in mediating kinetochore-microtubule (k-MT) attachment. To further prove the role of phosphorylated DAB2IP in establishing k-MT attachment, we measured the inter-KT distance in parental DAB2IP-deficient C4-2 Neo cells and wild-type DAB2IP- and DAB2IP 2A-expressing cells. In parental C4-2 Neo cells, the inter-KT distance between sister KTs was 1.03 ± 0.12 μM at metaphase. This distance increased to 1.22 ± 0.19 μM in C4-2 D2 cells, but decreased to about 1.03 ± 0.11 μM in C4-2 2 A cells (Fig. 7E, F).

We found that C4-2 cells expressing the DAB2IP 2A mutant, but not wild-type DAB2IP, exhibited increased aneuploidy (Supplementary Fig. S5C and S5D), which is highly correlated with carcinogenesis and aggressive cancer cells. To further determine the implications for tumorigenesis, we subcutaneously injected DAB2IP-deficient C4-2 parental cells and C4-2 cells expressing wild-type DAB2IP and DAB2IP 2A mutant into athymic nude mice and recorded the volume of tumors. The expression of wild-type DAB2IP totally abrogated tumor growth. However, the DAB2IP 2A mutant promoted PCa tumor growth (Fig. 7G, H). These data indicate that the phosphorylation of DAB2IP on the Thr531 and Thr546 sites is required for SAC maintenance and aneuploidy suppression and is essential to DAB2IP’s inhibitory effect on tumor development.

## Discussion

In this study, we found that tumor suppressor protein DAB2IP undergoes phosphorylation at Thr531 and Thr546 residues in its central region by Cdk1 during mitosis, and these phosphorylation sites are critical for activating the SAC and facilitating k-MT attachment during mitosis (Figs. 5 and 7). Our previous work showed that DAB2IP interacts with and activates PLK1 in mitosis [32]. PLK1 protein contains two C-terminal regulatory PBD motifs, which interact with the N-terminal kinase domain of PLK1; this intra-molecule binding is sufficient to inhibit its activity [34]. Crystallography studies revealed that PBD has a higher affinity for a number of proteins after “primed” phosphorylation at certain serine or threonine sites. These interactions between PBD and target proteins dock PLK1 to particular subcellular structures and contribute to the release and activation of PLK1 kinase domain [35]. We found that Thr phosphorylation of DAB2IP (Thr531 and Thr546) is essential for the DAB2IP-PLK1 interaction during mitosis. The phosphoryl-deficient DAB2IP (Thr531Ala and Thr546Ala) mutant does not affect PLK1’s localization on different mitotic apparatuses (data not shown), but it dramatically impairs the phosphorylation of PLK1 on Thr210 in its T-loop, which is required for the full activation of PLK1 (Fig. 6 and Supplementary Fig. S5). PLK1 is frequently considered an oncogenic protein due to its essential role in driving cell division in tumor cells. Constitutive expression of PLK1 in NIH 3T3 cells leads to the formation of oncogenic foci [36]. The expression of PLK1 has been observed to be higher in PCas than in the adjacent normal tissue, and its expression levels are linked with tumor grades [37]. However, by using an inducible knock-in mouse model, a recent study also showed that PLK1 acts as a tumor suppressor. PLK1 overexpression decreases cell proliferation because it disrupts proper mitotic progression. The same study reported delayed Kras- and Her2-induced mammary gland tumor development in PLK1-overexpressing mice. In patients, the expression of PLK1 is positively associated with good prognosis in specific breast cancer subtypes [38]. In our present study, we failed to select the stable PCa cell lines with constitutive expression of phospho-mimicking DAB2IP mutation (DAB2IP 2D, T531D/T546D). Sustained activation of PLK1 induced by phospho-DAB2IP mimic mutant may cause severe mitotic defects and, subsequently, cell death in PCa cells. In contrast, the DAB2IP mutant with alanine substitutions of the Thr531 and Thr546 residues increased chromosomal instability in PCa cells and promoted tumor growth in the PCa xenograft model (Fig. 7 and Supplementary Fig. S5). We also observed that N-terminus depletion helped with the phosphorylation of DAB2IP on its Thr sites (Supplementary Fig. S4C), which indicates that the N-terminal domain may block phosphoryl-sites of DAB2IP in interphase cells to avoid premature PLK1 activation. How the structure of DAB2IP controls Cdk1’s access to its Thr531 and Thr546 sites needs to be investigated further to fully clarify the spatiotemporal control of the PLK1 signal pathway in mitosis, through which DAB2IP tightly maintains chromosomal stability and inhibits carcinogenesis. It is worth noting that, even Cdk1 suppression inhibited mitotic DAB2IP phosphorylation; however, the CPR fragment can be almost equally phosphorylated in asynchronous HeLa cells as in mitotic cells (Supplementary Fig. S4C). Therefore, we cannot exclude the possibility that other Cdks also contribute to the phosphorylation and functions of DAB2IP in other cell cycle phases. The CPR region of DAB2IP is also essential for mediating its interaction with other signal pathways members, such as PI3K p85 [24, 25]. We found that Thr phosphorylation of DAB2IP (Thr531 and Thr546) does not affect the interaction between DAB2IP and PI3K p85 (data not shown).

Mps1 is recruited to kinetochores without the attachment of the microtubule from the mitotic spindle, and its kinase activity is critical for SAC signal initiation [4]. When localized to unattached kinetochores, Mps1 phosphorylates MELT motifs of KNL1 and provides a docking site for Bub1-Bub3 and BubR1-Bub3 to initiate a catalytic cycle involving Mad1, Mad2, and Cdc20 that eventually forms the MCC [7]. Mps1 and PLK1 share similar phosphorylation consensus motifs in substrates, including autophosphorylation sites of Mps1. PLK1-mediated phosphorylation of Mps1 stimulates Mps1 autophosphorylation and activity in vitro [12, 39]. Our study demonstrated that DAB2IP can facilitate Mps1 kinase activity in mitosis, and loss of DAB2IP or phosphoryl-deficient mutations impairs the mitotic autophosphorylation of Mps1 (Figs. 3 and 6). Consistent with the function of Mps1 in MCC initiation, the presence of DAB2IP promotes the binding of Mad2 and BubR1 with Cdc20 and the APC/C complex. On the other hand, loss of DAB2IP or the introduction of Thr531Ala and Thr546Ala mutations into DAB2IP contributes to the dissociation of the MCC from the APC/C, leading to premature SAC inactivation and APC/C activation (Fig. 1, Supplementary Figs. S1 and S5).

Compared to normal cells, cancer cells commonly exhibit a weakened SAC, which causes chromosome missegregation and aneuploidy as well as cancer development [40–42]. However, complete loss of the SAC is lethal even for tumor cells. Thus, because Mps1 is crucial for SAC activation, targeting Mps1 is an attractive antitumor strategy. Unlike taxol-related anti-microtubule agents, which induce prolonged mitotic arrest, targeting the SAC accelerates mitotic progression and generates a lethal level of chromosomal instability in cancer cells. Our study and studies by other groups demonstrated that aggressive PCa with decreased expression of DAB2IP is resistant to anti-microtubule agents. Here, we found that Cdk1-mediated phosphorylation of DAB2IP on Thr531 and Thr546 confers sensitivity to paclitaxel in PCa cells, which suggests that the increased cell killing from taxol-related agents in DAB2IP-abundant PCa cells stems from the cells being trapped in mitosis (Fig. 7). More importantly, our study showed that DAB2IP-deficient PCa cells have a greater sensitivity to Mps1 inhibitor than DAB2IP-proficient cells with a robust SAC (Fig. 4). Targeting Mps1 is a much easier route to minimizing interactions between the MCC and APC/C and to inducing misaligned chromosomes, followed by apoptosis in DAB2IP-deficient PCa cells (Fig. 4). Our findings provide a rationale for applying an anti-SAC drug, but not an anti-microtubule agent, against aggressive PCa with silenced DAB2IP gene expression.

In addition to the production of the MCC from unattached kinetochores, limiting the dissociation of the APC/C-MCC is also essential for SAC maintenance in mitosis. To date, several proteins have been identified as components of the MCC and as possible participants in checkpoint silencing, including PP1 [43], p31^comet^ [43, 44], UbcH10 [45], CUEDC2 [17], APC15 [20, 46, 47] and long noncoding RNA CRYBG3 [48]. Previous studies revealed that Cdc20 autoubiquitylation is necessary for MCC release. Moreover, proteasome inhibition leads to the accumulation of large amounts of MCC-APC/C during SAC activation [47, 49]. Uzunova et al. showed that continuous cycles of synthesis and degradation of Cdc20 during prometaphase promote rapid MCC turnover and SAC silencing when MCC production is stopped at the kinetochore [20]. APC15 is a subunit of the APC/C complex and is responsible for Cdc20 autoubiquitylation and proteasomal degradation during prometaphase, which triggers MCC disassembly [20]. In addition to APC15, p31^comet^ can also facilitate Cdc20 autoubiquitylation and MCC dissociation. In this study, we revealed a novel role that DAB2IP plays in SAC maintenance by stabilizing APC/C-MCC (Fig. 1). We found that DAB2IP can interact with Cdc20 through its PR domain and inhibit Cdc20 ubiquitylation and degradation during mitosis (Fig. 2). Therefore, DAB2IP negatively regulates APC/C-MCC turnover and slows the degradation of cyclin B1 and securin after nocodazole release (Fig. 1 and Supplementary Fig. S1). Gao et al. showed that CUEDC2 can be phosphorylated by Cdk1 and that this phosphorylation is necessary for binding with Cdc20 and contributes to MCC dissociation by disrupting the Mad2-Cdc20 interaction [17]. Contrarily, Cdk1-mediated phosphorylation of DAB2IP on Thr531 and Thr546 inhibits Cdc20 ubiquitylation during mitosis and blocks the disassembly of APC/C-MCC (Fig. 6). Considering that these phosphorylation sites on DAB2IP contributes to PLK1 activation, the PLK1-Mps1 signal pathway might play a determinate role in blocking the ubiquitylation of Cdc20 and the release of MCC from the APC/C complex. As described earlier, APC15 is essential for Cdc20 ubiquitylation and degradation in response to the SAC signal. Whether PLK1-Mps1 participates in APC15 activation warrants further investigation. It is worth noting that Dr. H. Yu’s group found that PLK1 can also directly phosphorylate Cdc20, which is sufficient to inhibit APC/C-Cdc20 activity via an MCC-independent mechanism. Whether Cdk1-mediated phosphorylation of DAB2IP facilitates Cdc20’s phosphorylation by PLK1 remains to be answered.

**Fig. 8:**
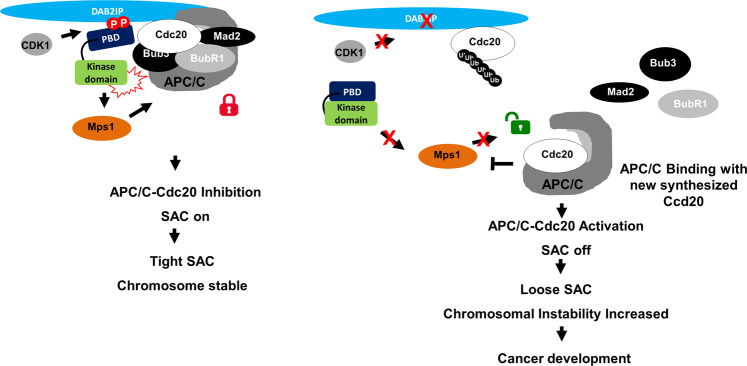
Proposed model of the role of DAB2IP’s phosphorylation in SAC maintenance and cancer development suppression. Cdk1 phosphorylates DAB2IP on its central region during mitosis. This phosphorylation provides a docking position for PLK1 and, meanwhile, activates the kinase activity of PLK1 and its downstream Mps1 kinase to promote the initiation of the MCC. Loss of expression or Cdk1-mediated phosphorylation of DAB2IP leads to a weakened SAC and increases chromosome missegregation and aneuploidy, which contributes to tumorigenesis and chemoresistance.

In summary, we propose the following model for DAB2IP’s role in mitosis (Fig. 8). Cdk1 phosphorylates DAB2IP on its central region during mitosis. This phosphorylation provides a docking position for PLK1 and, meanwhile, activates the kinase activity of PLK1 and its downstream Mps1 kinase to promote the initiation of the MCC. DAB2IP, as a component of APC/C-MCC, directly binds with Cdc20 through its PR and inhibits ubiquitylation and degradation of Cdc20 during prometaphase, which then blocks the disassembly of APC/C-MCC and inhibits premature SAC silencing. DAB2IP likely acts as a scaffold to promote PLK1 targeting of Cdc20. Loss of expression or Cdk1-mediated phosphorylation of DAB2IP leads to a weakened SAC and increases chromosome missegregation and aneuploidy, which contributes to tumorigenesis and chemoresistance. Targeting the SAC might be an attractive strategy against aggressive PCa with a weak SAC.

## Materials and methods

### Cell lines and treatment

C4-2, PC3, and DU145 PCa cells were maintained in a T medium (Invitrogen, Carlsbad, CA, USA) containing 5% fetal bovine serum (FBS) (HyClone, Hudson, NH, USA), 10 mM HEPES, 1 mM sodium bicarbonate, 100 U/ml penicillin, and 100 μg/ml streptomycin in a humidified incubator at 37 °C with 5% CO_2_. C4-2 D2 and control (C4-2 Neo) cells were generated from C4-2 cells as described previously (24), and human DAB2IP isoform 2 cDNA (1065a.a.) were stably expressed in C4-2 cells. C4-2 D2 2A mutant expressing clones were selected by G418 (Sigma, St Louis, MO, USA) at a concentration of 1000 μg/ml after 4 weeks. All C4-2 Neo, C4-2 D2, C4-2 D2 2A-1, and C4-2 D2 2A-2 cells were maintained in media including 500 μg/ml of G418. HeLa human cervical cancer cells were obtained from the American Type Culture Collection, and cells were maintained in a minimum essential medium containing 10% FBS and penicillin/streptomycin in a humidified incubator at 37 °C with 5% CO_2_. Cell lines were routinely tested by using Mycoplasma-free Mycoplasma Detection Kit (Beyotime, China). All of the cell lines were authenticated by using short tandem repeat (STR) profiling detection by the Cell Bank of Typical Culture Preservation Committee of the Chinese Academy of Sciences. Cells were treated with 50 ng/ml nocodazole (Sigma, St. Louis, MO, USA), 10 nM paclitaxel (Sigma, St. Louis, MO, USA), 20 μM MG132 (Sigma, St. Louis, MO, USA), 5 μM Cdk1 inhibitor RO-3306 (Selleckchem, Houston, TX, USA), or Mps1 inhibitor AZ3146 (0.25, 0.5 and 1 μM, Selleckchem, Houston, TX, USA) for indicated times. Cell transfection with small inhibitory RNA (siRNA) oligonucleotides or expression constructs of DAB2IP was performed by using Lipofectamine 3000 (ThermoFisher Scientific, Carlsbad, CA, USA). SiRNA oligonucleotides against DAB2IP were used as previously described [32].

To study Mps1 protein stability, we incubated DAB2IP-proficient and -deficient C4-2 and PC3 cells with CHX (Sigma, St Louis, MO, USA), and collected cells at indicated post-treatment time points. Cell lysates were subjected to immunoblot analysis to detect the protein level of Mps1.

### Plasmids

The HA-tagged and Myc-tagged Cdc20 plasmids were obtained from Addgene (Addgene, #11594 and #11593) [50]. The DAB2IP siRNA resistant expression plasmid and various DAB2IP full length and truncated expression plasmids were described previously [25, 32]. A sequence-verified open reading frame clone of PLK1 was cloned into a pCMV-HA expression plasmid. Point mutations for DAB2IP were generated by using a QuikChange II site-directed mutagenesis kit (Agilent, Santa Clara, CA, USA).

### Antibodies, immunoprecipitation, and immunofluorescence

The following primary antibodies were used in the present study: anti-DAB2IP, anti-BubR1 (Bethyl Laboratories, Montgomery, TX, USA); anti-Cyclin B1, anti-Hsp70, anti-PARP, anti-PLK1-pT210, anti-PLK1 (Cell Signaling Technology Danvers, MA, USA); anti-HA, anti-Cdc20 control mouse IgG and rabbit IgG (Santa Cruz Biotechnology, Dallas, TX, USA); anti-H3-pSer10 (Millipore, Bellerica, MA, USA); anti-α-tubulin, anti-Flag (M2), anti-β-actin (Sigma-Aldrich, St. Louis, MO, USA); anti-Mad2 and anti-Cdc27 (BD Biosciences, San Jose, CA, USA); anti-Securin (MBL International, Woburn, MA, USA); anti-Mps1 (Ancam, Cambridge, MA, USA); and anti-Mps1-pT676 (Thermo Fisher Scientific, Waltham, MA, USA). The secondary antibodies used for immunofluorescence were Alexa Fluor 488 goat anti-mouse IgG, Alexa Fluor 488 goat anti-rabbit IgG, Alexa Fluor 488 goat anti-human IgG, Alexa Fluor 568 goat anti-mouse IgG, and Alexa Fluor 568 goat anti-rabbit IgG (Invitrogen Carlsbad, CA, USA).

C4-2 Neo, D2, and DAB2IP-deficient, and -abundant PC3 HeLa cells were synchronized with nocodazole at prometaphase, and mitotic shake-off cells lysates were incubated with anti-DAB2IP, anti-Cdc20, anti-Cdc27, or anti-Mps1 antibodies and protein A/G sepharose overnight. The sepharose beads were washed with lysate buffer three times and resuspended in an sodium dodecyl sulfate (SDS)-PAGE loading buffer for immunoblot analysis using indicated antibodies.

For immunofluorescence analysis, cells undergoing the different treatment regimens were plated on 35 mm dishes with coverslips, fixed in 4% paraformaldehyde/PBS for 30 min, permeabilized in 0.5% Triton X-100/PBS (phosphate-buffered saline) for 15 min, and blocked in 5% bovine serum albumin for 30 min. The samples were incubated with anti-α-tubulin (1:1000), anti-Crest (1:10,000), and anti-BubR1 (1:500) antibodies for 3 h at room temperature, washed three times in PBS (5 min each), and incubated with Alexa Fluor 488 and 568 secondary antibodies (1:1000) for 1 h. The cells were washed in PBS three times (5 min each) and mounted using VECTASHIELD with 4', 6-diamidino-2-phenylindole (DAPI) (Vector Laboratories, Burlingame, CA, USA). Images were taken under a fluorescence microscope (Axio Imager M2, Carl Zeiss, Thornwood, NY, USA) with AxioVision SE64 Rel.4.8 software (Carl Zeiss, Thornwood, NY, USA). BubR1 protein and phosphorylated Mps1-T676 intensity at kinetochores was quantified by drawing a circle closely along the Crest signaling. The intensity values from the peak three continuous stacks were subtracted from the background from adjacent areas and averaged. The intensity was normalized against the related Crest signal intensity. To quantify inter-kinetochore distances, all images were acquired as a Z-stack with 0.4 μm spacing using a ×63 objective.

### MTT

Exponentially growing C4-2 Neo, D2, and D2 2A cells were transfected in 96-well plates (2000 cells per well). Cells were treated with indicated doses of paclitaxel and AZ3146 for 48 h and assayed for viability using the previously described MTT assay [51].

### In vitro kinase assays

For in vitro Mps1 kinase assay, immunoprecipitated endogenous Mps1 protein from C4-2 Neo, C4-2 D2 mitotic shake-off cells (cells were treated with 50 ng/ml nocodazole for 16 h) and asynchronous cells were incubated in Mps1 kinase reaction buffer (20 mM HEPES at pH 7.4, 100 mM KCl, 10 mM MgCl2, 1 mM EDTA, 0.5 mM DTT, 5% glycerol, 10 μM ATP and 0.17 μM γ-^32^P ATP) with MBP as substrates. The reaction took place in an incubator for 30 min at 30 °C and was stopped by adding SDS sample buffer. After the kinase reaction, samples were subject to SDS-polyacrylamide gel electrophoresis analysis. Incorporation of ^32^P was determined by a Typhoon 9410 Imager (GE Healthcare Life Sciences) [32].

### Chromosome spread assays

C4-2 Neo, D2 and, D2 2A cells were treated with 100 ng/ml colcemid (Irvine Scientific, Santa Ana, CA, USA) for 6 h. Cells were harvested and hypotonically swollen in pre-warmed 0.075 M KCl for 13 min at 37 °C. Cells were prefixed through directly adding 1 ml freshly made Carnoy’s fixative solution (methanol: acetic acid 3:1) into the hypotonic cell suspension for 5 min. Cells were then fixed in freshly made Carnoy’s fixative solution. Cells were dropped onto warmed glass slides and dried overnight. Slides with spread cells were stained with 5% Giemsa for 15 min at room temperature, gently rinsed with running water, air dried, and mounted. Slides were visualized via microscope (BX51, Olympus, Tokyo, Japan) and images were recorded with SPOT Advanced software (SPOT Imaging Solutions, Sterling Heights, MI, USA).

### Ubiquitylation detection

HeLa and 293T cells were single or co-transfected with 2.0 μg HA-ubiquitin, 1.0 μg Flag-DAB2IP (or Flag-DAB2P 2A or 2D mutants), and Myc-Cdc20. After 42 h of transfection, 10 μM MG132 was added to the cells for 6 h and cells were lysed by using a lysate buffer (2% SDS, 150 mM NaCl, 10 mM Tris-HCl, pH 7.5, 2 mM sodium orthovanadate, 50 mM sodium fluoride, and protease inhibitors). Lysates were immunoprecipitated with anti-Myc antibody and analyzed by immunoblotting using anti-HA, anti-Myc and, anti-Flag antibodies.

### Mouse xenograft tumors study

C4-2 Neo, D2, D2 2A-1, and D2 2A-2 cells (7 × 10^6^ in 100 μl serum-free RPMI-1640 containing Matrigel, 1:1, v/v) were injected subcutaneously into the right flank of male athymic nude mice (6–8 weeks old). Tumor volume measurements were recorded once a week and calculated as follows: $L \times W \times H \times 0.5236$. The data are presented as the mean ± SEM. Investigators were blinded to the group allocation during the experiment. The size of tumors was regularly measured and no randomization was used. The mouse study was approved by the laboratory animal welfare ethics review at Soochow University.

## Conclusion

Tumor suppressor DAB2IP can be phosphorylated by Cdk1 during mitosis, and the phosphorylation of DAB2IP activates the PLK1-Mps1 pathway, inhibits the ubiquitylation of Cdc20 in prometaphase, and blocks the premature release of APC/C-MCC. Depletion or loss of Cdk1-mediated phosphorylation of DAB2IP destabilizes the MCC, impairs the SAC, and increases chromosome missegregation and subsequent CIN, which contributes to tumorigenesis. Targeting the SAC to cause lethal CIN is an attractive therapeutic strategy against DAB2IP-deficient aggressive PCa.

## Supplementary information

Supplemental Material

## Data Availability

All data generated during the current study are available upon request.
