# Supplementary material for: Mitotic phosphorylation of tumor suppressor DAB2IP maintains spindle assembly checkpoint and chromosomal stability through activating PLK1-Mps1 signal pathway and stabilizing mitotic checkpoint complex
Source: Oncogene. 2021 Nov 13;41(4):489–501. doi: 10.1038/s41388-021-02106-8 (PMC8782720; doi:10.1038/s41388-021-02106-8)
Supplement: Supplementary file 1 — Supplemental Material [file 41388_2021_2106_MOESM1_ESM.pdf]

Supplementary Information

Mitotic phosphorylation of tumor suppressor DAB2IP maintains spindle assembly checkpoint and chromosomal stability through activating PLK1-Mps1 signal pathway and stabilizing mitotic checkpoint complex

Lan Yu[1,2†], Yue Lang[3,†], Ching-Cheng Hsu[1†], Wei-Min Chen[1], Jui-Chung Chiang[1], Jer-Tsong Hsieh[4,5,6], Michael D. Story[1,5], Zeng-Fu Shang[1,3,*], Benjamin P.C. Chen[1,5,*], Debabrata Saha[1,5*]

[1]Department of Radiation Oncology, University of Texas Southwestern Medical Center, Dallas, TX 75390, USA

[2]Suzhou Digestive Diseases and Nutrition Research Center, The Affiliated Suzhou Hospital of Nanjing Medical University, Suzhou 215008, China

[3]State Key Laboratory of Radiation Medicine and Protection, School of Radiation Medicine and Protection, Medical College of Soochow University, Collaborative Innovation Center of Radiation Medicine of Jiangsu Higher Education Institutions, Soochow University, Suzhou, 215123, China

[4]Department of Urology, University of Texas Southwestern Medical Center, Dallas, TX 75390, USA

[5]Simmons Comprehensive Cancer Center, University of Texas Southwestern Medical Center, Dallas, TX 75390, USA

[6]Department of Oncology, National Taiwan University Hospital, National Taiwan University College of Medicine, Taipei 10048, Taiwan

[*]To whom correspondence should be addressed.

Zeng-Fu Shang. Tel: +1 214 648 1188; Fax: +1 214 648 5995; Email: ziyu_620@163.com; zengfu.shang@utsouthwestern.edu.

Benjamin Chen. Tel: +1 214 648 1263; Fax: +1 214 648 5995; Email: benjamin.chen@utsouthwestern.edu.

Debabrata Saha. Tel: +1 214 648 7750; Fax: +1 214 648 5995; Email: debabrata.saha@utsouthwestern.edu.

[†]These authors contributed equally to the work as first authors.

Fig. S1

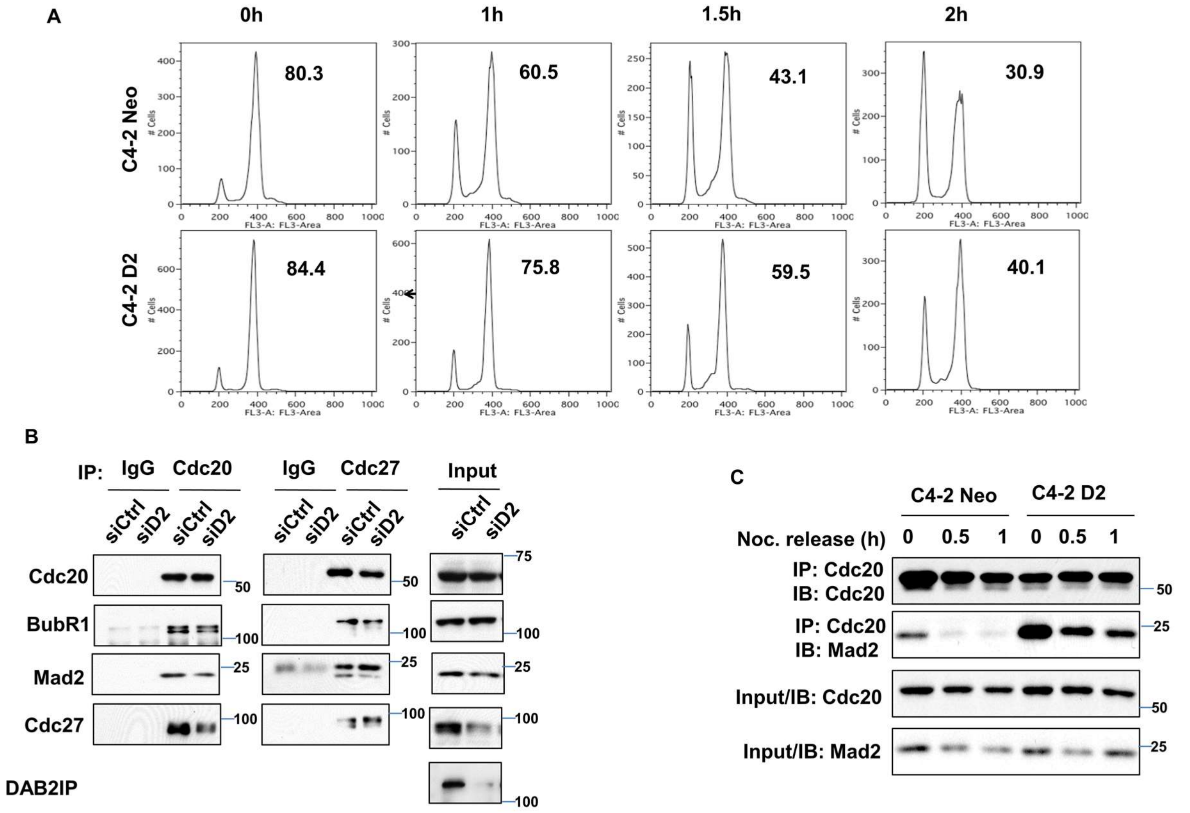

Supplemental Figure 1. DAB2IP prevents premature mitotic exit and maintains MCC stability. **A** DAB2IP-proficient (D2) and DAB2IP-deficient (Neo) C4-2 cells were synchronized in prometaphase using nocodazole and then released into fresh media. Cells were collected at the indicated times after release. The cell cycle distribution of PCa cells at indicated times was analyzed by flow cytometry. **B** Cdc20 and APC/C complex were immunoprecipitated from nocodazole blocked DAB2IP-knockdown HeLa and its control prometaphase cell lysates. The amounts of Mad2 and BubR1 binding with Cdc20 and Cdc27 were determined by immunoblotting. **C** C4-2 Neo and D2 cells were synchronized in prometaphase by nocodazole and released into fresh media. The Cdc20 was immunoprecipitated at the indicated time points after nocodazole releasing. The amount of Mad2 binding with Cdc20 was visualized by immunoblotting. Whole cell lysates were analyzed using the indicated antibodies.

Fig. S2

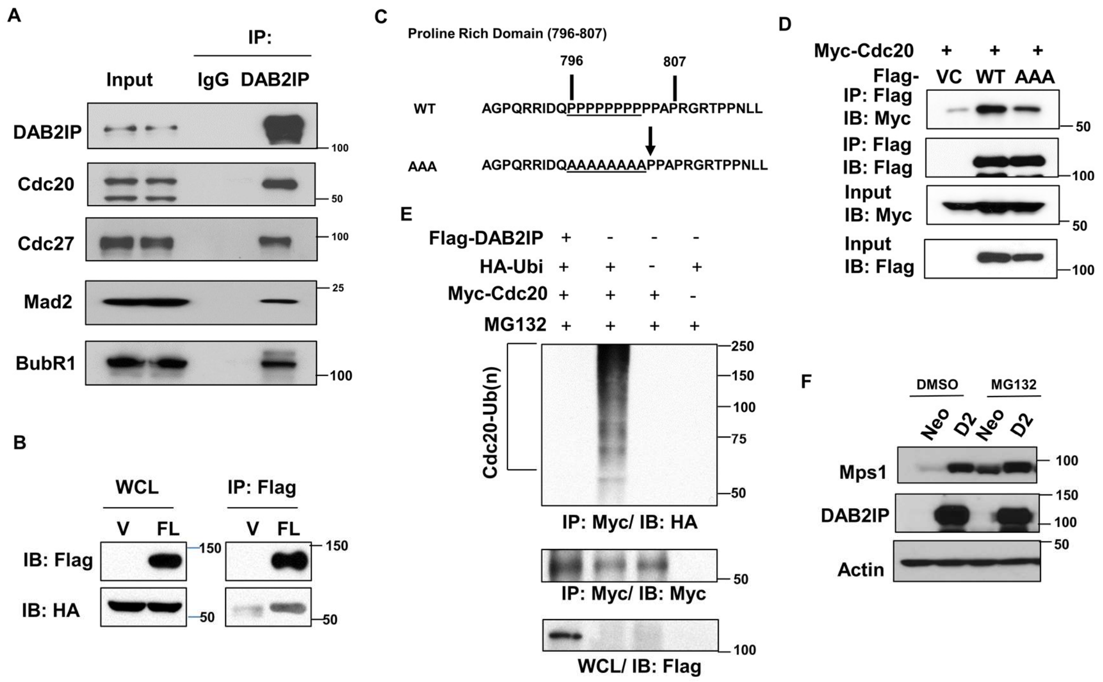

Supplemental Figure 2. DAB2IP interacts with Cdc20, and inhibits the ubiquitylation of Cdc20 in prometaphase. **A** HeLa cells were synchronized at prometaphase by nocodazoel, and the mitotic cells were collected by the shake-off method. Cells lysates were immunoprecipitated with anti-DAB2IP or IgG antibodies, and the interaction with Cdc20, Cdc27, BubR1 and Mad2 were analyzed by immunoblotting. **B** Flag-DAB2IP, Flag empty vector and HA-Cdc20 were transfected into 293T cells. 293T cells were then synchronized at prometaphase and the mitotic cells were collected by the shake off method. Cells lysates were immunoprecipitated with anti-Flag with antibody. The signal of HA and Flag were determined by immunoblotting. **C** The cluster of eight proline in DAB2IP is underlined and was replaced with alanine to generate DAB2IP AAA mutant. **D** Flag tagged-DAB2IP, -DAB2IP AAA (8P>A), empty vector and Myc-Cdc20 were transfected into HeLa cells. The cells were then treated with 50 ng/ml nocodazole for 16 hours, and mitotic cell lysates were immunoprecipitated with anti-Flag antibody. The Myc signal was examined by immunoblotting. **E** Myc-Cdc20 and HA-Ubi were co-transfected with Flag-DAB2IP or empty vector in 293T cells. Cells were harvested at 6 hours after MG132 (10 μM) treatment. Cdc20 was immunoprecipitated using anti-Myc antibody. Co-IP products were denatured and analyzed by immunobloting using anti-HA, and anti-Myc antibodies. Equal loading of whole cell lysates were determined by immunoblotting using anti-Flag antibody. **F** Proteasome inhibitor MG132 recovered the expression levels of Mps1 in DAB2IP-deficient C4-2 Neo cells.

Fig. S3

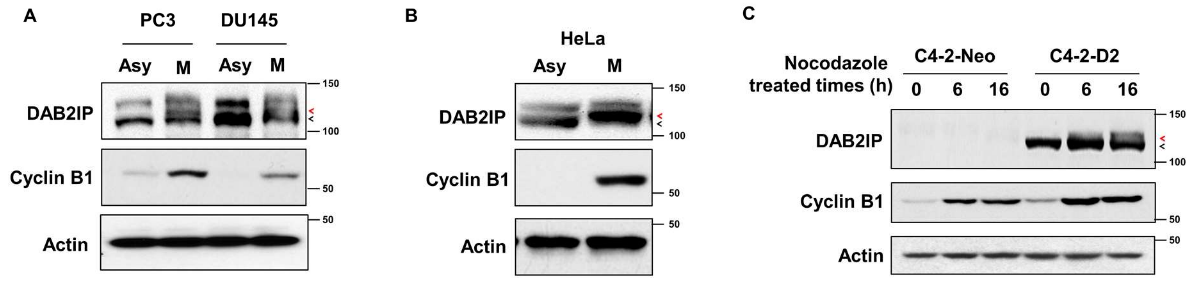

Supplemental Figure 3. DAB2IP is phosphorylated in mitosis. (**A**, **B** and **C**) The expression pattern of DAB2IP, Cyclin B1 and Actin in mitotically arrested (M, nocodazole-induced) and asynchronous (Asy) PC3, DU145 (**A**), HeLa (**B**), C4-2 Neo and C4-2 D2 (**C**) cells were determined by using immunoblotting.

Fig. S4

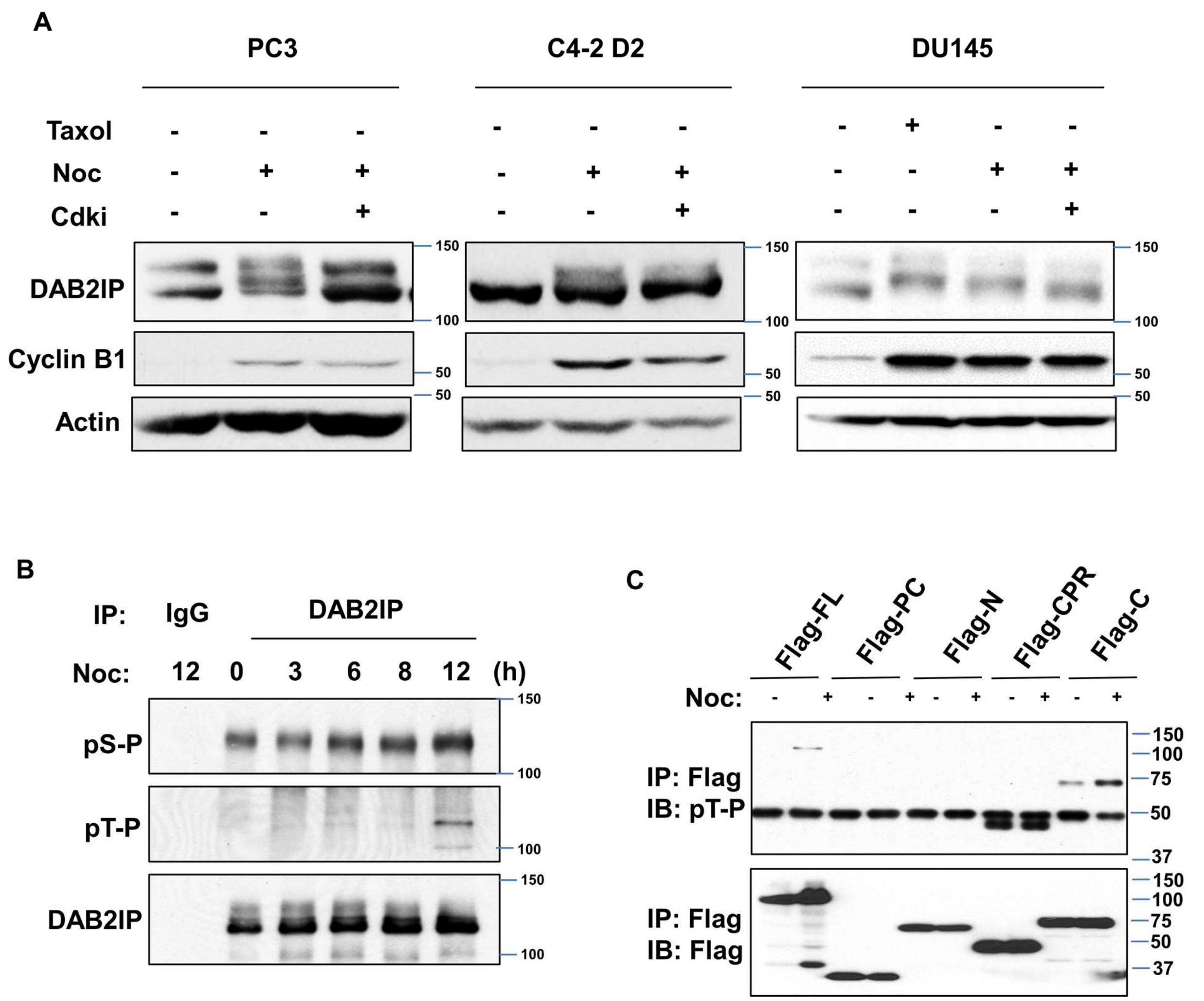

Supplemental Figure 4. DAB2IP is phosphorylated by Cdks on its CPR region in mitosis. (**A**) PC3, C4-2 D2 and DU145 cells were arrested at prometaphase and treated with Cdk1 inhibitor RO-3306 along with nocodazole for 2 hours. The expression of DAB2IP, Cyclin B1 and Actin was determined by immunoblotting. (**B**) C4-2 D2 cells were treated with nocodazole from indicated time points, and DAB2IP was immunoprecipitated from C4-2 D2 cell lysates in various cell cycle phases. The phosphorylation of DAB2IP was detected by using antibodies which can specifically recognize of Cdk1 targeting sites on pS-P and pT-P. (**C**) Full length and various truncated cDNA constructs of DAB2IP were transfected into HeLa cells, and these cells were synchronized in prometaphase by nocodazole. Cell lysates were immunoprecipitated by using anti-Flag antibody. The phosphorylation of DAB2IP on its TP sites was determined by immunoblotting.

Fig. S5

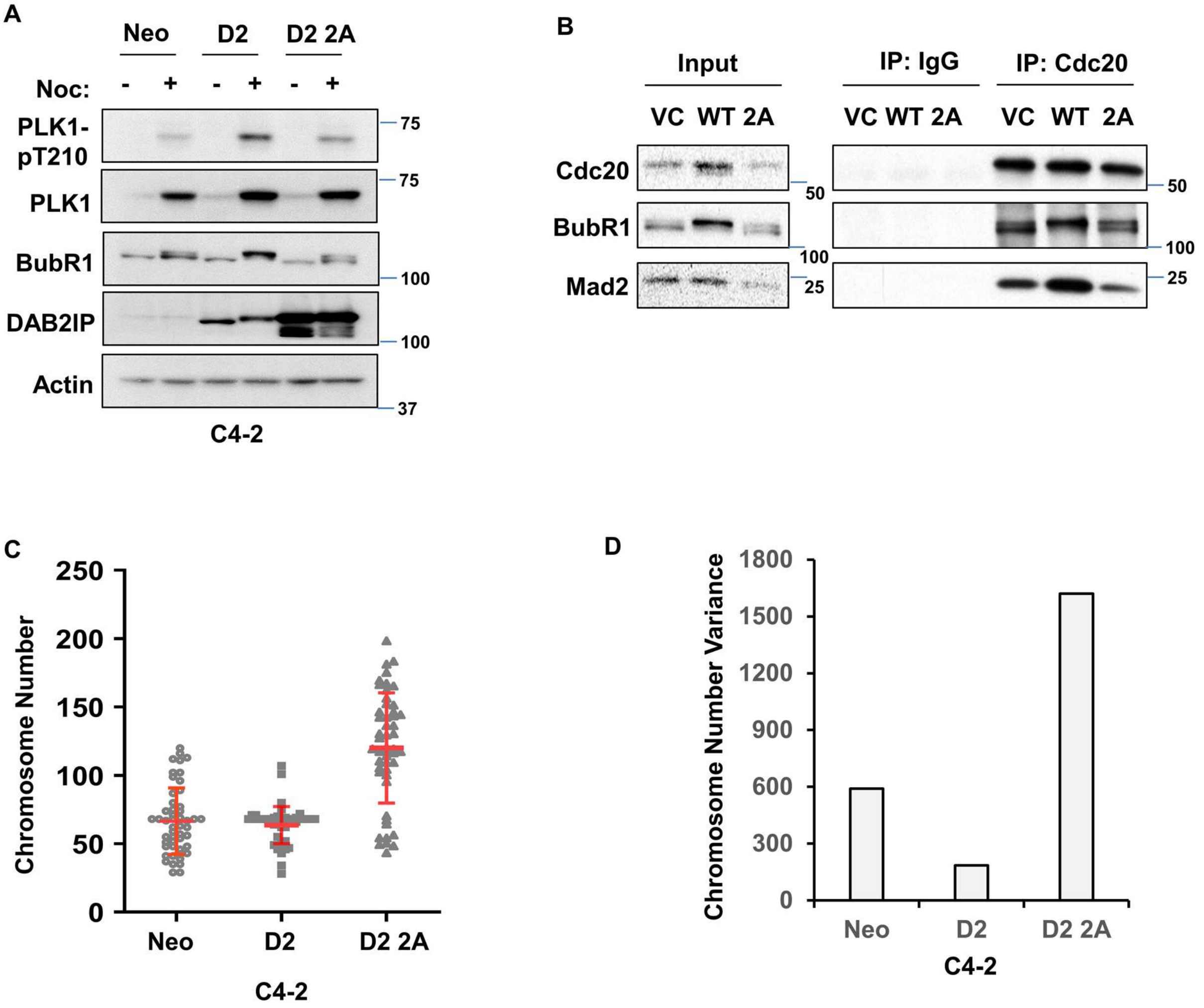

Supplemental Figure 5. Phosphorylation of DAB2IP at its Thr531 and Thr546 sites activates PLK1-Mps1 signal pathway and maintains chromosomal stability. (**A**) Immunoblotting analysis of levels of PLK1-pT210, PLK1, BubR1 in mitotically arrested or asynchronized C4-2 Neo, D2 and D2 2A cells. (**B**) Cdc20 was immunoprecipitated from nocodazole blocked C4-2 D2, D2 2A and Neo prometaphase cells lysates. The amounts of Mad2 and BubR1 binding with Cdc20 were determined by immunoblotting. (**C**) Chromosome numbers from individual metaphase spreads of C4-2 Neo, D2 and D2 2A cells. Each point represents an individually analyzed cell. (**D**) Chromosome number variance of each cell lines shown in (**C**) was calculated by ANOVA single factor testing, using Microsoft Excel.
